# Antimicrobial Activity and Synergy Investigation of *Hypericum scabrum* Essential Oil with Antifungal Drugs

**DOI:** 10.3390/molecules26216545

**Published:** 2021-10-29

**Authors:** Layal Fahed, Marc El Beyrouthy, Naïm Ouaini, Véronique Eparvier, Didier Stien, Sara Vitalini, Marcello Iriti

**Affiliations:** 1 UPR 2301, Institut de Chimie des Substances Naturelles, CNRS, 1 Avenue de la Terrasse, 91198 Gif-sur-Yvette, France; layal.fahed@ul.edu.lb (L.F.); veronique.eparvier@cnrs.fr (V.E.); didier.stien@cnrs.fr (D.S.); 2 Natural Sciences Department, Faculty of Sciences II, Lebanese University, B.P. 90656, Fanar 15216, Lebanon; 3 Department of Agricultural Sciences, Holy Spirit University of Kaslik, Jounieh 1200, Lebanon; naimouaini@usek.edu.lb; 4 Laboratoire de Biodiversité et Biotechnologies Microbiennes (LBBM), Observatoire Oceanologique, UPMC Univ Paris 06, CNRS, Sorbonne Universités, 66650 Banyuls-sur-Mer, France; 5 Department of Agricultural and Environmental Sciences, Università degli Studi di Milano, 20133 Milano, Italy; sara.vitalini@unimi.it; 6 Phytochem Laboratory, Department of Agricultural and Environmental Sciences, Università degli Studi di Milano, 20133 Milano, Italy; 7 National Interuniversity Consortium of Materials Science and Technology, 50121 Firenze, Italy; 8 BAT Center-Interuniversity Center for Studies on Bioinspired Agro-Environmental Technology, Università degli Studi di Napoli Federico II, 80055 Portici, Italy

**Keywords:** *Hypericum scabrum*, essential oil, chemical composition, antimicrobial activity, synergy

## Abstract

The chemical composition of Lebanese *Hypericum scabrum* essential oil (EO) was analyzed by gas chromatography (GC) and gas chromatography-mass spectrometry (GG-MS). Its antimicrobial activity was evaluated by determining its minimal inhibitory concentrations (MICs) against a Gram-negative and a Gram-positive bacterium, one yeast, and five dermatophytes. *H. scabrum* EO was most active on filamentous fungi (MIC values of 32–64 µg/mL). Synergy within the oil was investigated by testing each of the following major components on *Trichophyton rubrum*: *α*-pinene, limonene, myrcene, *β*-pinene and nonane, as well as a reconstructed EO. The antifungal activity of the natural oil could not be reached, meaning that its activity might be due, in part, to minor constituent(s). The interactions between *H. scabrum* EO and commercially available antifungals were assessed by the checkerboard test. A synergistic effect was revealed in the combination of the EO with amphotericin B.

## 1. Introduction

Antimicrobial resistance is jeopardizing public health, and is thus turning into an urgent global concern. Resistance occurrence is the result of random genetic events, such as mutations and horizontal gene transfers that enable microbial genomes to evolve [1], while its persistence and spread in the ecosystem is favored by the selective pressure of antimicrobial agents [2,3].

One approach to avoid the emergence of resistant strains is combination therapy that consists of associating several antimicrobial agents. The chance of having mutants that are resistant to agents with independent mechanisms of action is extremely small. Resistance is thus less likely to develop [4]. Such associations could also be used to improve the therapy efficiency when they result in synergistic effects. Another advantage of such combinations is that lower doses are used [5], reducing the side effects and cost of the treatment [6]. In this field, associations between plant essential oils and classic antimicrobials have proven their efficiency [7,8].

Plants belonging to the genus *Hypericum* are well known for their therapeutic benefits. The most popular species is *Hypericum perforatum* (St John’s Wort). Its extract is one of the best-selling herbal medicines in the world. It is used to treat mild-to-moderate depression [9,10].

*Hypericum scabrum* L. (Hypericaceae) has traditionally been, in different parts of the world, the basis of the medical treatment of many ailments, such as hemorrhoids [11], body heat [12], stomach ache, diarrhea [13], sun burn, and skin lesions [14].

Although extracts and essential oils (EOs) of *H. scabrum* have been investigated and reported to have relevant biological activities [15,16,17,18], our Lebanese EO differs significantly from those obtained elsewhere. It was, therefore, interesting to investigate its chemical composition and to evaluate its antimicrobial activity against selected microorganisms responsible for skin infections in humans. In fact, in the topical route of application that is often used traditionally, the EOs are administered in direct contact with the pathogen, and the adverse effects that they could cause when administered systemically become limited. In the present work, we demonstrate that the very good antifungal activity of the Lebanese *H. scabrum* does not originate from its major constituents, either alone or combined. Since the strong antifungal potential was due to minor components, we embarked upon investigating the potential of this oil to exacerbate the activity of conventional antimicrobials.

## 2. Results and Discussion

### 2.1. Chemical Analysis

**Figure 1:**
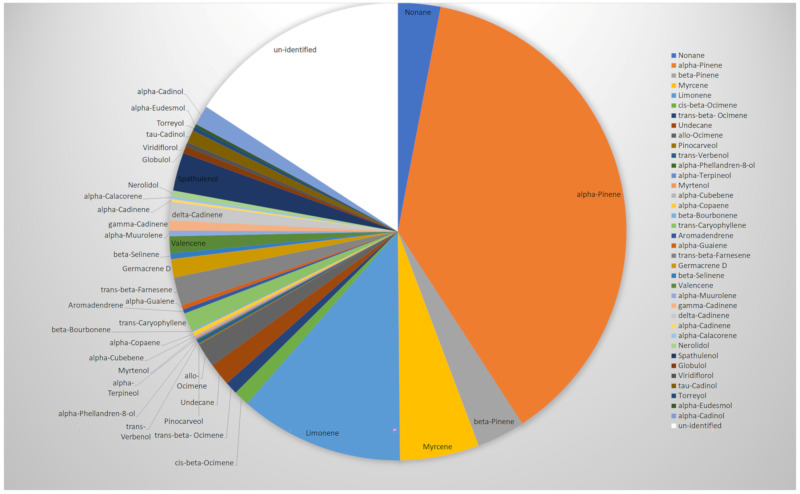
Pie chart representing the chemical composition of *Hypericum scabrum* essential oil (EO).

The hydrodistillation of the aerial parts of *H. scabrum* yielded 0.6% essential oil. Thirty-seven compounds, representing 84.2% of the whole oil, were identified (Table 1; Figure 1).

**Figure 2:**
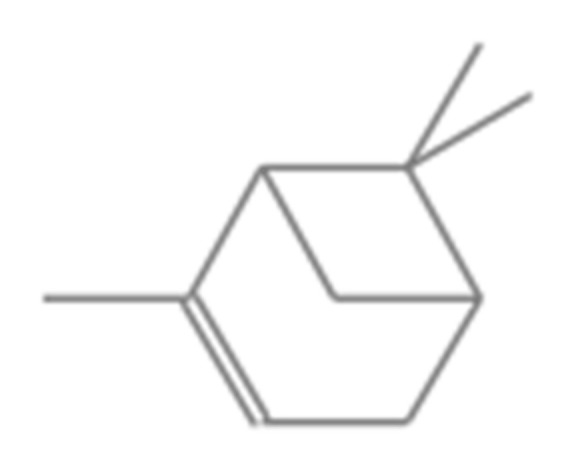
Chemical structure of *α*-pinene.

**Figure 3:**
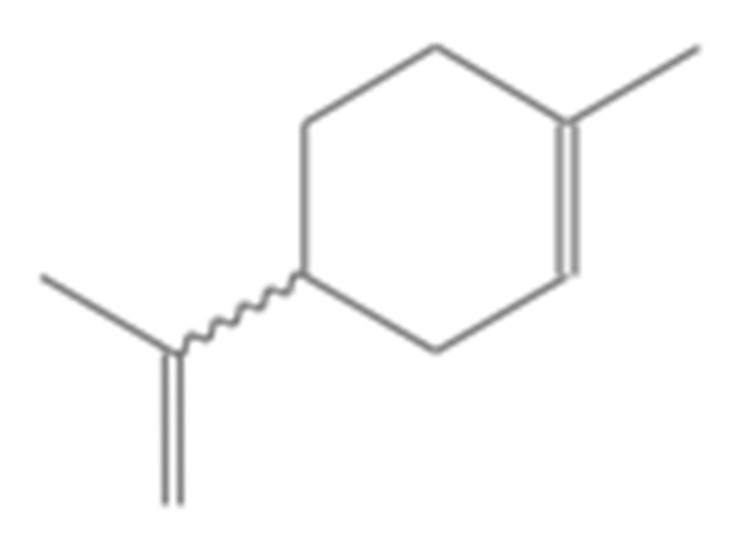
Chemical structure of limonene.

**Figure 4:**
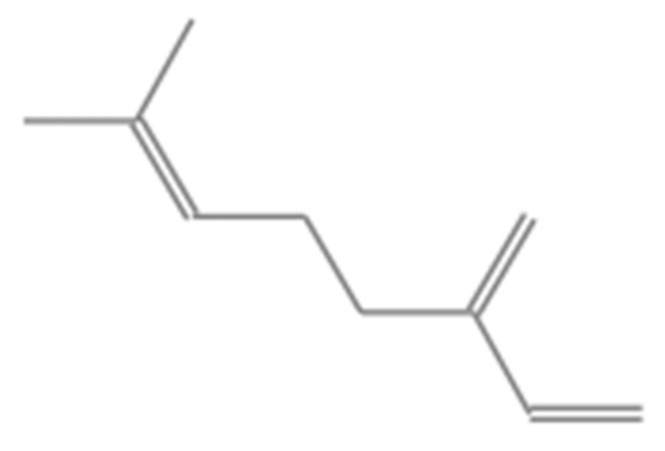
Chemical structure of myrcene.

**Figure 5:**
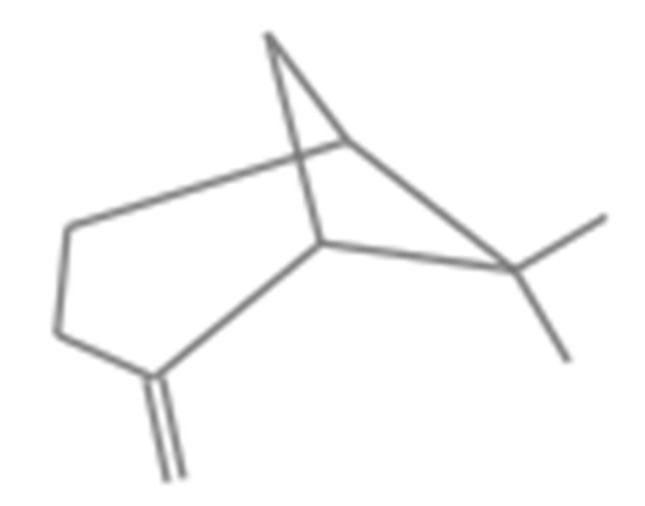
Chemical structure of *β*-pinene.

**Figure 6:**
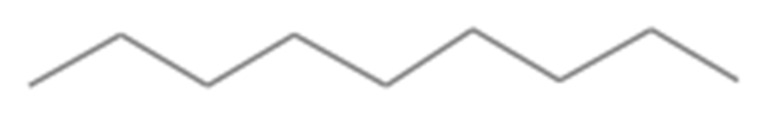
Chemical structure of nonane.

*α*-Pinene (Figure 2) was the most abundant compound (37.8%), followed by limonene (11.6%) (Figure 3), myrcene (5.6%) (Figure 4), *β*-pinene (3.4%) (Figure 5), and nonane (3%) (Figure 6). It is noteworthy that *α*-pinene was also the major compound of the previously studied *H. scabrum* essential oils from the following different regions of the world: Iran [19], Turkey [20], Tajikistan [21], and Uzbekistan [22]. This does not apply to the other major compounds, which were sometimes minorly present or even totally absent in these other samples.

### 2.2. Antimicrobial Activity

The results of the antimicrobial activity of *H. scabrum* EO are presented in Table 2. EOs with minimal inhibitory concentrations (MICs) of 128 µg/mL and below are generally considered active [23,24].

The oil was inactive on *Pseudomonas aeruginosa* and *Staphylococcus aureus* (MIC > 512 µg/mL). Conversely, *H. scabrum* EO presented significant antifungal activity against filamentous dermatophytic fungi (MIC values of 32–64 µg/mL, whereas *Candida albicans* was weakly sensitive (MIC of 512 µg/mL).

Dermatophytes were the most sensitive microorganisms, which is a result that can be explained by the functional role of volatile organic compounds (VOCs), since the majority of pathogens infecting plants are fungi, most of them being filamentous [25,26].

In an attempt to shed light on the origin of the antifungal activity, we individually tested the major components and a reconstructed EO against *Trichophyton rubrum* (Table 3). The artificial EO was prepared using the major compounds in pure form, in the same proportions as in the EO, whereas the minor compounds were replaced by DMSO (38.6%).

*α*-Pinene, limonene, myrcene, *β*-pinene, and nonane showed no significant activity (MIC of 512 µg/mL). The reconstructed EO exhibited moderate activity (256 µg/mL), which points to a probable synergy between the major compounds. It is, however, less active than the natural EO (64 µg/mL), indicating that minor constituents contribute to the overall activity of the EO.

The interaction between *H. scabrum* and antifungal drugs was tested by the checkerboard assay on *T. rubrum*, and the results are given in Table 4. A synergistic effect (FICI 0.5) was observed by combining the EO with amphotericin B. When the EO was added at a concentration of 32 µg/mL, the MIC of amphotericin B was lowered from 4 to 0.125 µg/mL, thus the combined use of *Hypericum scabrum* essential oil with amphotericin B could reduce the amount of amphotericin B by 32 for the same antifungal activity. On the other hand, the associations with fluconazole and griseofulvin were moderately synergistic (FICI 0.6).

## 3. Materials and Methods

### 3.1. Plant Material

Aerial parts of *Hypericum scabrum* were collected in June 2013 from Mzaar Kferdebian, Mount Lebanon (33°59′38″ N 35°50′5″ E) at an altitude of 1818 m. The plant identification was based on ‘Nouvelle flore du Liban et de la Syrie’ by Paul Mouterde [27]. Air drying of the plant materiel was performed in a shady place for two weeks at room temperature.

### 3.2. Essential Oil Extraction

The extraction of the essential oil was carried out by hydrodistillation for three hours using a Clevenger-type apparatus according to the European Pharmacopoeia, 1997 [28].

### 3.3. GC Analyses

Analytical gas chromatography was performed on a Thermo Electron Corporation gas chromatograph fitted with a flame ionization detector (FID), a DB-5 MS capillary column (30 m × 0.25 mm) with 0.1 µm film thickness or a fused silica HP Innowax polyethylene glycol capillary column (50 m × 0.20 mm, film thickness 0.20 µm). Helium was the carrier gas (0.7 mL/min). The column temperature was initially set to 35 °C before being gradually increased to 85 °C at 5 °C/min, held for 20 min at 85 °C, raised to 300 °C at 10 °C/min and finally held for 5 min at 300 °C. Diluted 1 µL samples (1/100, *v/v*) were injected at 250 °C manually and in the splitless mode. Flame ionization detection (FID) was performed at 310 °C.

### 3.4. GC–MS Analyses

The GC–MS analyses were performed using an Agilent 6890 gas chromatograph coupled with 5975 mass detector. The 7683 B auto sampler injected 1 µL of each diluted oil sample (1/100, *v/v*). A fused silica capillary column DB-5 MS (30 m × 0.25 mm internal diameter, film thickness 0.1 µm) or a fused silica HP Innowax polyethylene glycol capillary column (50 m × 0.20 mm, film thickness 0.20 µm) was used. Helium was the carrier gas (0.7 mL/min). The oven temperature program was identical to that described above (c.f. *3.*3 GC Analysis). The mass spectra were recorded at 70 eV with an ion source temperature of 310 °C and a transfer line heated to 320 °C. The acquisition was recorded in full scan mode (50–400 m/z).

### 3.5. Qualitative and Quantitative Analysis

Most constituents were identified by gas chromatography by comparing their retention indices (RI) with those from the literature [29,30] or with those of authentic compounds obtained from Sigma-Aldrich (Beirut-Lebanon, and Paris-France). The retention indices were determined relatively to a homologous series of n-alkanes (C_8_ to C_24_) analyzed under the same operating conditions. Concomitantly, their mass spectra on both columns were compared with those provided in the NIST and Wiley 275 libraries, which are our home-made libraries constructed with pure compounds and EOs of known composition or with mass spectra from the literature [29,31]. The relative concentration of each component was calculated based on the GC peak areas without using correction factors.

### 3.6. Antimicrobial Activity

Gram-negative bacterial strain *Pseudomonas aeruginosa* CIP 82118, Gram-positive bacterial strain *Staphylococcus aureus* ATCC 29213, yeast *Candida albicans* ATCC 10231, and the clinical isolates of dermatophytes *Trichophyton rubrum* SNB-TR1, *Trichophyton mentagrophytes* SNB-TM1, *Trichophyton soudanense* SNB-TS1, *Trichophyton violaceum* SNB-TV1 and *Trichophyton tonsurans* SNB-TT1 [32] were used in this study.

The antimicrobial activity of the EOs was measured using a broth microdilution method according to the Clinical and Laboratory Standards Institute (CLSI) guidelines [33,34,35,36,37]. The essential oil and its major components were diluted in DMSO and were tested at concentrations ranging from 16 to 512 µg/mL. The microplates were incubated at 37 °C for 24 h for bacteria, 48 h for yeasts, and 5 days for dermatophytes. The minimal inhibitory concentrations (MIC) refer to the lowest concentrations that prevent visible microbial growth (Table 2 and Table 3). Oxacillin and gentamicin (0.03–16 µg/mL) were used as reference antibiotics, while itraconazole (0.03–16 µg/mL) and fluconazole (0.125–64 µg/mL) were used as positive controls for the antifungal assays. The antimicrobial standards were purchased from Molekula–Dorset, UK and the pure terpenes from Sigma-Aldrich, France.

### 3.7. Synergy Test by Microdilution Checkerboard Technique

This test is performed in a 96-well microplate and is based on the microdilution method. In particular, two-fold dilution series of the essential oil are made in the vertical direction on the plate while those of the antimicrobial agent are made in the horizontal direction of the same plate, in order to have a fixed amount of the first agent in each row and column and a decreasing amount of the second agent [38,39]. The essential oil was tested at concentrations ranging from 256 to 8 µg/mL. Antimicrobial agents were tested at concentrations ranging from 16 to 0.03 µg/mL for griseofulvin and amphotericin B, and from 64 to 0.125 µg/mL for fluconazole.

The quantitative assessment of the interactions was based on the calculation of fractional inhibitory concentrations (FICs) defined as the ratio of the MIC of the combinations of the two components to the MIC of the essential oil or the drug alone, and the FIC index (FICI), which is the sum of the two FICs. The results were interpreted as follows:

FICI ≤ 0.5; synergistic effect > 0.5 and ≤ 2.0; additive effect > 2; antagonistic effect as proposed by Shin and Lim (2004) [40].

## 4. Conclusions

Our results showed that *H. scabrum* EO exhibited interesting antifungal activity. This activity could not be attributed to one major constituent of the oil. It clearly resulted from the interaction of several constituents, including minor ones. In addition, its synergistic interaction with amphotericin B, and the moderately synergistic association with fluconazole and griseofulvin, suggested that combinations between this EO and conventional antifungal agents may lead to new, more potent formulations with less dose-related toxicity.

## Figures and Tables

**Table 1:** Chemical composition of *Hypericum scabrum* essential oil (EO).

RI ^a^	RI ^b^	Compounds	CAS No	Molecular Formula	(%)
901	-	Nonane	111-84-2	C_9_H_20_	3.0
938	1076	*α*-Pinene	80-56-8	C_10_H_16_	37.8
980	1118	*β*-Pinene	127-91-3	C_10_H_16_	3.4
993	1174	Myrcene	123-35-3	C_10_H_16_	5.6
1030	1203	Limonene	138-86-3	C_10_H_16_	11.6
1045	1269	*cis*-*β*-Ocimene	3338-55-4	C_10_H_16_	1.1
1050	1253	*trans*-*β*-Ocimene	3779-61-1	C_10_H_16_	0.9
1100	1490	Undecane	1120-21-4	C_11_H_24_	1.6
1134	1493	*allo*-Ocimene	673-84-7	C_10_H_16_	1.7
1138	1664	Pinocarveol	5947-36-4	C_10_H_16_O	0.1
1152	1683	*trans*-Verbenol	1820-09-3	C_10_H_16_O	0.2
1168	1697	*α*-Phellandren-8-ol	1686-20-0	C_10_H_16_O	0.1
1189	1706	*α*-Terpineol	98-55-5	C_10_H_18_O	0.1
1196	1804	Myrtenol	515-00-4	C_10_H_16_O	0.1
1352	1466	*α*-Cubebene	17699-14-8	C_15_H_24_	0.1
1377	1497	*α*-Copaene	3856-25-5	C_15_H_24_	0.3
1385	1533	*β*-Bourbonene	5208-59-3	C_15_H_24_	0.1
1415	1612	*trans*-Caryophyllene	87-44-5	C_15_H_24_	1.3
1437	1628	Aromadendrene	109119-91-7	C_15_H_24_	0.3
1439	1650	*α*-Guaiene	3691-12-1	C_15_H_24_	0.3
1455	1689	*trans*-*β*-Farnesene	18794-84-8	C_15_H_24_	2.0
1477	1726	Germacrene D	23986-74-5	C_15_H_24_	1.3
1485	1711	*β*-Selinene	17066-67-0	C_15_H_24_	0.4
1495	1740	Valencene	4630-07-3	C_15_H_24_	1.2
1500	1740	*α*-Muurolene	10208-80-7	C_15_H_24_	0.4
1515	1776	*γ*-Cadinene	39029-41-9	C_15_H_24_	0.7
1525	1772	*δ*-Cadinene	483-76-1	C_15_H_24_	1.3
1526	1773	*α*-Cadinene	24406-05-1	C_15_H_24_	0.2
1541	1918	*α*-Calacorene	38599-17-6	C_15_H_20_	0.2
1566	2050	Nerolidol	142-50-7	C_15_H_26_O	0.4
1577	2008	Spathulenol	6750-60-3	C_15_H_24_O	2.7
1585	2098	Globulol	489-41-8	C_15_H_26_O	0.5
1591	2104	Viridiflorol	552-02-3	C_15_H_26_O	0.3
1640	2188	*T*-Cadinol	5937-11-1	C_15_H_26_O	0.9
1645	2145	Torreyol	19435-97-3	C_15_H_26_O	0.3
1649	2188	*α*-Eudesmol	473-16-5	C_15_H_26_O	0.2
1650	2256	*α*-Cadinol	481-34-5	C_15_H_26_O	1.4
		Total identified			84.2

^(a)^ Retention index on a DB-5MS colum. ^(b)^ Retention index on an HP Innowax column.

**Table 2:** Antimicrobial activity (minimal inhibitory concentration (MIC) in µg/mL) of *Hypericum scabrum* EO.

Pathogens	*S. aureus* ATCC 29213	*C. albicans* ATCC 10231	*P. aeruginosa* CIP 82118	*T. rubrum* SNB-TR1	*T. mentagrophytes* SNB-TM1	*T. soudanense* SNB-TS1	*T. violaceum* SNB-TV1	*T. tonsurans* SNB-TT1
*Hypericum scabrum* EO	> 512	512	> 512	64	32	32	32	64
Reference compounds	1 ^a^	1 ^b^	0.25 ^c^	2 ^b^	1 ^d^	2 ^b^	2 ^b^	4 ^b^

^a^ Oxacillin; ^b^ fluconazole; ^c^ gentamicin; ^d^ itraconazole.

**Table 3:** Antifungal activity (MIC in µg/mL) of the major constituents of *Hypericum scabrum* EO and a reconstructed EO from the major constituents on *Trichophyton rubrum*.

	Nonane	*α*-Pinene	*β*-Pinene	Myrcene	Limonene	Reconstructed EO (61.4%)
*T. rubrum* SNB-TR1	512	512	512	512	512	256

**Table 4:** Combined effects of *Hypericum scabrum* essential oil and conventional antimicrobial drugs on *Trichophyton rubrum*.

	Combination	EO: Fluconazole	EO: Griseofulvin	EO: Amphotericin B
EO	MIC_a_ MIC_c_ FIC	64 32 0.5	64 32 0.5	64 32 0.5
Drug	MIC_a_ MIC_c_ FIC	2 0.25 0.125	1 0.0625 0.0625	4 0.125 0.03
	FICI	0.6	0.6	0.5

MIC_a_: MIC of the EO or the drug alone (in µg/mL); MIC_c_: MIC of the EO or the drug at the relative proportion displaying the highest synergy (in µg/mL); FIC: fractional inhibitory concentration; FICI: FIC index.

## Data Availability

Not applicable.
